# Increasing incidence of group B streptococcus neonatal infections in the Netherlands is associated with clonal expansion of CC17 and CC23

**DOI:** 10.1038/s41598-020-66214-3

**Published:** 2020-06-12

**Authors:** Dorota Jamrozy, Merijn W. Bijlsma, Marcus C. de Goffau, Diederik van de Beek, Taco W. Kuijpers, Julian Parkhill, Arie van der Ende, Stephen D. Bentley

**Affiliations:** 1 Wellcome Sanger Institute, Wellcome Genome Campus, Hinxton, UK; 2 Department of Neurology, Amsterdam Neuroscience, Amsterdam University Medical Center, University of Amsterdam, Amsterdam, Netherlands; 3 Department of Immunopathology, Sanquin Research and Landsteiner Laboratory of the Academic Medical Center, University of Amsterdam, Amsterdam, Netherlands; 4 Department of Paediatric Haematology, Immunology and Infectious Diseases, Emma Children’s Hospital, Amsterdam University Medical Center, Amsterdam, Netherlands; 5 Department of Medical Microbiology, Amsterdam Infection and Immunity, Amsterdam University Medical Center, University of Amsterdam, Amsterdam, Netherlands; 6 Netherlands Reference Laboratory for Bacterial Meningitis, Center of Infection and Immunity Amsterdam, Amsterdam University Medical Center, Amsterdam, Netherlands; 7 Present Address: Department of Veterinary Medicine, University of Cambridge, Cambridge, UK

**Keywords:** Bacterial genes, Pathogens

## Abstract

Group B streptococcus (GBS) is the leading cause of neonatal invasive disease worldwide. In the Netherlands incidence of the disease increased despite implementation of preventive guidelines. We describe a genomic analysis of 1345 GBS isolates from neonatal (age 0–89 days) invasive infections in the Netherlands reported between 1987 and 2016. Most isolates clustered into one of five major lineages: CC17 (39%), CC19 (25%), CC23 (18%), CC10 (9%) and CC1 (7%). There was a significant rise in the number of infections due to isolates from CC17 and CC23. Phylogenetic clustering analysis revealed that this was caused by expansion of specific sub-lineages, designated CC17-A1, CC17-A2 and CC23-A1. Dating of phylogenetic trees estimated that these clones diverged in the 1960s/1970s, representing historical rather than recently emerged clones. For CC17-A1 the expansion correlated with acquisition of a new phage, carrying gene encoding a putative cell-surface protein. Representatives of CC17-A1, CC17-A2 and CC23-A1 clones were identified in datasets from other countries demonstrating their global distribution.

## Introduction

Group B streptococcus (GBS), also known as *Streptococcus agalactiae*, is a commensal bacterium that colonizes the human gastrointestinal and genital tracts^1^. It is also an opportunistic pathogen and a leading cause of neonatal invasive disease worldwide^2^. GBS disease in neonates occurs predominantly during the first 90 days of life and the most common clinical syndromes are septicaemia and meningitis^3,4^. The infection is defined as either early-onset disease (EOD, days 0–6) or late-onset disease (LOD, days 7–89)^5^. EOD results from intrapartum GBS transmission from a colonized mother whereas routes of GBS acquisition in LOD are less well defined and can involve vertical, nosocomial or community transmission^6^.

Since the 1990s many high-income countries have implemented policies for prevention of neonatal invasive GBS disease involving intrapartum antibiotic prophylaxis (IAP), which is effective in reducing the incidence of EOD^6^. Subsequently, the rates of EOD have declined substantially in a number of countries^7–10^. However, the challenge of reducing the burden of GBS disease in newborns remains. IAP has no effect on the incidence of LOD rates, which have remained stable or increased^7,9^. Moreover, increased incidence of both EOD and LOD has been recently reported in some countries despite their implementation of national disease prevention guidelines, indicating that reassessment of their current prevention strategies is needed^4,11^. This includes the Netherlands, the UK and Ireland, where IAP is offered based on the presence of clinical risk factors, in contrast to a more widely implemented culture-based screening for GBS carriage during pregnancy^12^. The reasons for this rise in disease incidence are poorly understood. Possible explanations include changes in the host, medical practice or the pathogen’s population. In the Netherlands, a shift in the prevalence of certain GBS clonal complexes (CC) was reported following the implementation of IAP policy in 1999, with an increase in the number of cases caused by isolates representing CC17^11^. Isolates from CC17 are overrepresented among invasive GBS isolates from infections in newborns and are particularly associated with meningitis and LOD cases with CC17 often referred to as a hypervirulent clone^3^.

GBS isolates are commonly genotyped using multi-locus sequence typing (MLST), which allows to group GBS isolates into sequence types (STs) that can then be clustered into major CC groups. MLST revealed that the GBS population has a relatively low genetic diversity and most isolates belong to a small number of dominant STs^13^. This is replicated when inferring species population structure of GBS using whole genome sequence-based phylogeny, which splits GBS into well-resolved lineages that represent major CCs^14^. Genomic analysis allows a higher level of isolate discrimination, offering further insights into population structure of individual GBS lineages. Genome-based study of a geographically diverse GBS collection reported that most isolates within major CCs cluster into one or two dominant clonal groups, suggesting that the contemporary GBS population structure has been shaped by evolutionary bottlenecks and clonal expansion events^14^. Beyond molecular epidemiology, genomic data also offers insights into mechanisms of GBS evolution and host adaptation. In this study we examined the genomes of 1345 GBS isolates from neonatal invasive disease in the Netherlands collected between 1987 and 2016 to investigate changes in pathogen population during a previously reported rise in disease incidence^11^.

## Results

### Genotypic characteristics of Dutch GBS from neonatal invasive disease

Clustering of isolates using hierBAPS was highly concordant with MLST and CC assignment correlated with GBS population structure revealed by a core genome-based phylogeny (Supplementary Fig. S1). Of the 1345 GBS isolates, the majority (n = 1321, 98%) clustered into five dominant human-associated lineages. The most common group was CC17 (526/1345; 39%), followed by CC19 (332/1345; 25%), CC23 (239/1345; 18%), CC10 (127/1345; 9%), and CC1 (97/1345; 7%). The remaining isolates (24/1345; 2%) were assigned to the hierBAPS bin group, a polyphyletic cluster of low-frequency genotypes. It consisted of isolates representing 7 different STs (Supplementary Data S1). Four of the STs were phylogenetically distinct and represented CC22, CC26, CC103 and CC130. The remaining three STs in the bin group were ST23, ST88 and ST280 that formed a monophyletic cluster most closely related to the CC23 clade.

The majority of GBS isolates were derived from EOD (830/1345; 62%). EOD isolates were most common in CC1 (79/97; 81%), followed by CC10 (97/127; 76%), CC23 (173/239; 72%) and CC19 (216/332; 65%). CC17 was represented by a significantly lower proportion of EOD isolates (248/526, 47%) in comparison with all other CCs (pairwise chi-squared test with Bonferroni correction; CC17: CC1, *p* value = 1.03 × 10^−8^; CC17: CC10, *p* value = 5.77 × 10^−8^; CC17: CC19, *p* value = 4.25 × 10^−6^; CC17: CC23, *p* value = 1.32 × 10^−9^). Consequently, CC17 had the highest proportion of LOD isolates (278/526, 53%), followed by CC19 (116/332, 35%), CC23 (66/239, 28%), CC10 (30/127, 24%) and CC1 (18/97, 19%)

This was also reflected in proportion of different CCs within each disease onset group. More than half of LOD isolates were CC17 (278/515, 54%), followed by CC19 (116/515, 23%), CC23 (66/515, 13%), CC10 (30/515, 6%) and CC1 (18/515, 3%). In contrast, CC17 represented only around a third of EOD isolates (248/830, 30%), followed by CC19 (216/830, 26%), CC23 (173/830, 21%), CC10 (97/830, 12%) and CC1 (79/830, 10%).

A key marker for monitoring the molecular epidemiology of GBS disease is the capsular polysaccharide (CPS) serotype. The most common CPS serotype in this collection was III (817/1345; 61%), followed by Ia (262/1345; 19%). Other serotypes were represented by up to 6% of isolates, which included Ib (84/1345; 6%), II (76/1345; 6%), V (64/1345; 5%), IV (29/1345; 2%), VI (5/1345; 0.4%), IX (4/1345; 0.3%) and VII (2/1345; 0.1%) (Supplementary Data S1). All but one CC17 isolate carried CPS serotype III (525/526; 99%), which was also observed in the majority of CC19 isolates (278/332; 84%). All but two CC23 isolates carried serotype Ia (237/239; 99%). Isolates from CC1 and CC10 revealed high serotype heterogeneity with eight and six different serotypes, respectively (Supplementary Data S1).

Recent studies reported a rise in the prevalence of antimicrobial resistance among clinical GBS isolates, in particular to macrolides, which was associated mostly with CC1 and CC19^15–17^. In our collection, 8% of isolates carried at least one macrolide-lincosamide-streptogramin (MLS) resistance gene (*ermB*, *ermTR*, *mefA*, *msrD*, *lsaC*, *lsaE* or *lnuB*; Supplementary Data S1). Carriage of MLS resistance genes was highest in CC1 (19/97; 20%), followed by CC19 (30/332; 9%), CC17 (43/526; 8%), CC23 (12/239; 5%) and CC10 (5/127; 4%). Also of concern is the emergence of GBS with reduced susceptibility to beta-lactams, the first line antibiotics for IAP and treatment of GBS infections^17^. Here, we identified two isolates with mutations in the *pbp2x* gene that were previously associated with reduced susceptibility to beta-lactams (T394I and Q557E)^18^, both representing CC19. Human-associated GBS isolates often show a high prevalence of tetracycline resistance genes due to clonal expansion of GBS lineages that acquired mobile elements carrying *tet* determinants^14^. In this study, 85% of all isolates carried at least one tetracycline resistance gene (*tetM*, *tetL*, *tetO* or *tetW*; Supplementary Data S1). Prevalence of *tet* genes varied between CCs and was highest in CC17 (504/526, 96%), followed by CC23 (221/239, 92%), CC19 (272/332, 82%), CC1 (72/97, 74%) and CC10 (61/127, 48%).

### Increasing incidence of GBS neonatal infections involved expansion of specific CC17 and CC23 clones

**Figure 1:**
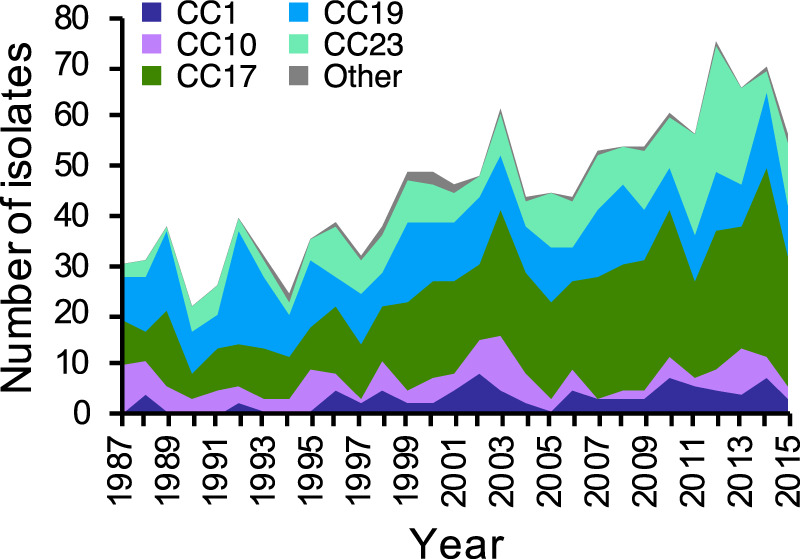
Number of invasive GBS isolates from neonatal infections submitted to the national Reference Laboratory per year stratified by CC. The exact number of isolates from each CC in each year is available in Supplementary Data S1. Number of isolates received in 2016 was excluded from the trend analysis as study sampling ended in May of that year.

The number of invasive GBS isolates submitted to the Reference Laboratory between 1987 and 2015 increased significantly over time (two-sided Mann-Kendall trend test, *p* value = 1.19 × 10^−7^; Fig. 1), reflecting a rise in the incidence of neonatal invasive GBS EOD and LOD in the Netherlands during the study period (Supplementary Fig. S2). This was mainly a result of a significant rise in a number of cases caused by isolates from CC17 and CC23 (two-sided Mann-Kendall trend test, *p* value <2.22 × 10^−16^, *p* value = 4.65 × 10^−6^, respectively). Between 1987 and 1995, the dominant GBS lineage each year was predominantly CC19. In the mid-1990s CC17 began to outcompete CC19 to become the dominant CC each year from 1996 onwards. Isolates from CC23 were less prevalent than CC19 initially but it became the second most common lineage from 2009. The increase in CC17 isolates was significant among both EOD and LOD cases (two-sided Mann-Kendall trend test, *p* value = 2.26 × 10^−6^ and *p* value = 1.58 × 10^−5^, respectively) (Supplementary Fig. S3). CC23 isolates showed a more significant increase among EOD than LOD cases (two-sided Mann-Kendall trend test, *p* value = 2.55 × 10^−5^ and *p* value = 0.008, respectively) (Supplementary Fig. S3). There were no significant changes in the frequency of isolates from CC10 and CC19 (two-sided Mann-Kendall trend test, *p* = 0.45 and *p* = 0.85, respectively), but a minor rise in CC1 (two-sided Mann-Kendall trend test, *p* value = 0.00076).

**Figure 2:**
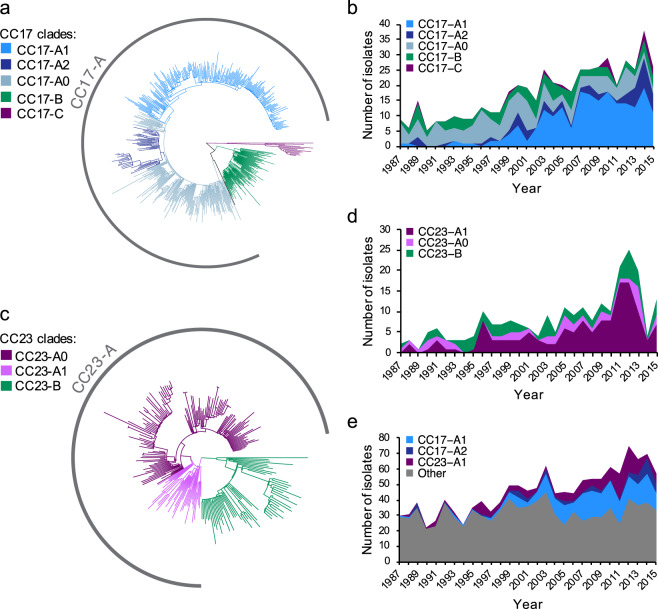
Phylogeny and temporal changes in population structure of CC17 and CC23 lineages. (**a**) Mid-point rooted maximum likelihood phylogenetic tree of CC17. Branches are coloured according to phylogenetic clade assignment. (**b**) Number of isolates from CC17 over time stratified by clade assignment. (**c**) Mid-point rooted maximum likelihood phylogenetic tree of CC23. Branches are coloured according to phylogenetic clade assignment. (**d**) Number of isolates from CC23 over time stratified by clade assignment. (**e**) Contribution of isolates from CC17-A1, CC17-A2 and CC23-A1 clades to the number of invasive GBS isolates from neonatal infections submitted each year. ‘Other’ – GBS isolates representing all other genotypes. The exact number of isolates in each year is available in Supplementary Data S1. Isolates from 2016 were excluded from trend analyses as study sampling ended in May of that year.

To better understand the temporal trends of GBS lineages, in particular the increase in CC17 and CC23 isolates, we investigated the population structure of each CC to identify intra-lineage clusters that changed significantly in prevalence over time. For this, we reconstructed and then partitioned a phylogeny of each CC. The majority of CC17 isolates (428/526, 81%) belonged to a single dominant clade defined here as CC17-A (Fig. 2). The number of isolates from this clade increased significantly over time (two-sided Mann-Kendall trend test, *p* value = 1.19 × 10^−7^). The CC17-A clade was partitioned further, which revealed that the increasing temporal trend was associated with expansion of two distinct clones, a larger cluster CC17-A1 (217/526; 41% [of all CC17 isolates]) and a minor cluster CC17-A2 (57/526; 11% [of all CC17 isolates]) (Fig. 2). The remaining CC17-A population was defined as CC17-A0. Only CC17-A1 and CC17-A2 showed a significant increase in a number of isolates over time (two-sided Mann-Kendall trend test, *p* = 1.19 × 10^−7^ and *p* = 0.0006, respectively), with CC17-A1 largely responsible for the overall rise in the number of cases due to CC17 (Fig. 2). CC17-A1 isolates were infrequent between 1987 and 1998 but started to increase in 1999, which led to CC17-A1 becoming a dominant CC17 clone by 2007. The occurrence of CC17-A1 isolates increased significantly amongst cases of both EOD and LOD (two-sided Mann-Kendall trend test, *p* = 3.1 × 10^−5^, *p* = 1.19 × 10^−7^, respectively) (Supplementary Fig. S4). Similarly to CC17, the majority of CC23 isolates (177/239, 74%) belonged to a single clade CC23-A (Fig. 2), which showed a significant increase in the number of isolates over time (two-sided Mann-Kendall trend test, *p* = 8.7 × 10^−6^). A further phylogenetic clustering of CC23-A revealed that this was due to an expansion of a sub-cluster CC23-A1 (137/239; 57% [of CC23 isolates]; Fig. 2). After grouping of isolates by disease onset, the increasing CC23-A1 trend was more significant for EOD than LOD cases (two-sided Mann-Kendall trend test, *p* = 6.79 × 10^−6^ and *p* = 0.026, respectively) (Supplementary Fig. S4). Overall, the increase in the number of cases caused by isolates representing CC17-A1, CC17-A2 and CC23-A1 was responsible for the general rise in isolate submissions over time with the largest contribution from the CC17-A1 clone (Fig. 2).

**Figure 3:**
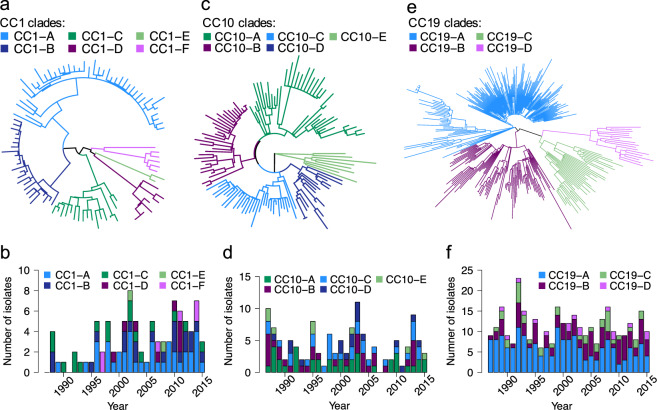
Phylogeny and temporal changes in population structure of CC1, CC10 and CC19 lineages. (**a**) Mid-point rooted maximum likelihood phylogenetic tree of CC1. Branches are coloured according to phylogenetic clade assignment. (**b**) Number of isolates from CC1 over time stratified by clade assignment. (**c**) Mid-point rooted maximum likelihood phylogenetic tree of CC10. Branches are coloured according to phylogenetic clade assignment. (**d**) Number of isolates from CC10 over time stratified by clade assignment. (**e**) Mid-point rooted maximum likelihood phylogenetic tree of CC19. Branches are coloured according to phylogenetic clade assignment. (**f**) Number of isolates from CC19 over time stratified by clade assignment. The exact number of isolates in each year is available in Supplementary Data S1. Isolates from 2016 were excluded from trend analyses as study sampling ended in May of that year.

Phylogenetic clustering was also performed for CC1, CC10 and CC19 to gain insights into their population structure and check for potential intra-lineage temporal trends. The phylogenetic tree of CC1 was partitioned into six clusters, while CC10 revealed five clusters but they showed no significant temporal changes (Fig. 3). CC19 clustered into four phylogenetic groups (Fig. 3). Isolates from the largest cluster CC19-A (183/332; 55% [of CC19 isolates]), showed a drop over time (two-sided Mann-Kendall trend test, *p* = 0.01), which was offset by a minor increase in the number of isolates from cluster CC19-B (two-sided Mann-Kendall trend test, *p* = 0.03) (Fig. 3).

### CC17-A1 and CC23-A1 are not recently emerged clones

To investigate how the recent rise in frequency of isolates representing the CC17-A1 and CC23-A1 clones correlated with the phylogenetic history of CC17 and CC23, we inferred time-calibrated Bayesian phylogenies of each CC. The estimated mutation rates were 8.51 × 10^−7^ (95% confidence interval [CI] 8.1 × 10^−7^ – 8.92 × 10^−7^) and 6.21 × 10^−7^ (95% CI 5.69 × 10^−7^ – 6.73 × 10^−7^) substitutions per nucleotide site per year for CC17 and CC23, respectively.

**Figure 4:**
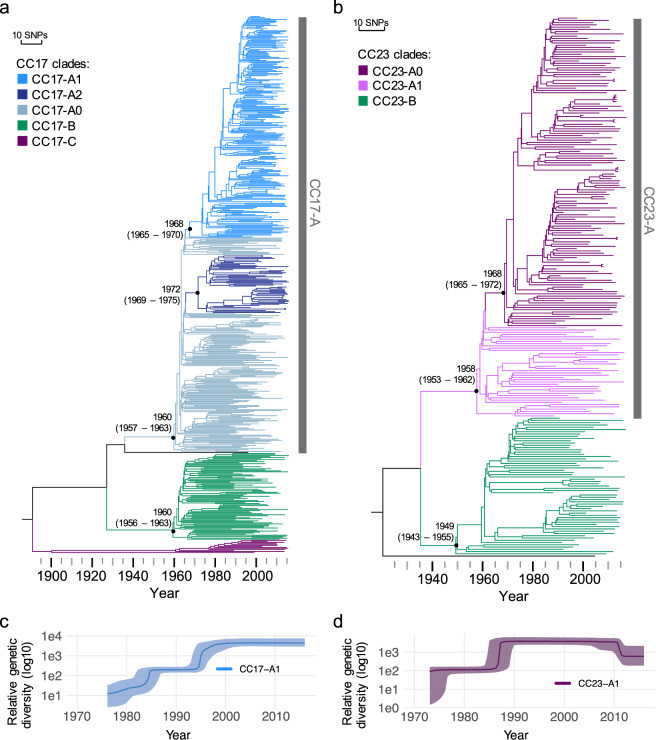
Evolutionary history of CC17 and CC23. (**a**) Bayesian maximum clade credibility tree of CC17. Tree branches are coloured in accordance with the CC17 clade assignment. (**b**) Bayesian maximum clade credibility tree of CC23. Tree branches are coloured in accordance with the CC23 clade assignment. (**c**) Bayesian coalescent skyline plot for CC17-A1 and (**d**) CC23-A1. Plot lines represent the median relative genetic diversity, over time. Band around each line represents the upper and lower 95% highest posterior density intervals.

For CC17 it was estimated that clades CC17-A and CC17-B emerged in parallel, around the turn of the 1950s and 1960s (Fig. 4). This was also the estimated time of emergence for the CC23-A clade while CC23-B emerged a decade earlier, around the turn of the 1940s and 1950s (Fig. 4). This parallel GBS clade divergence between the 1950s and 1970s is supported by the epidemiological history of GBS, which emerged at the time as a leading cause of infant invasive disease^19^. It is also in agreement with a previous study describing the emergence of adapted GBS clones in the mid-20^th^ century^14^. The time of the most recent common ancestor (TMRCA) for CC17-A1 was estimated around 1968 (95% highest posterior density [HPD] 1965-1970). Similarly, the CC23-A1 clade diverged around 1968 (95% HPD 1965–1972). Therefore, the expanding clusters represent historical genotypes rather than recently emerged clones.

We also estimated the demographic changes over time for CC17-A1 and CC23-A1 using the Bayesian skyline plot model. The CC17-A1 clone showed a sharp rise in the relative genetic diversity (RGD) around the mid-1990s, making its past population changes highly concordant with the epidemiology of CC17-A1 isolates in our collection (Fig. 4). In contrast, the CC23-A1 clone experienced the most recent RGD increase around the mid-1980s (Fig. 4).

### The CC17-A1 and CC23-A1 clones are globally distributed

**Figure 5:**
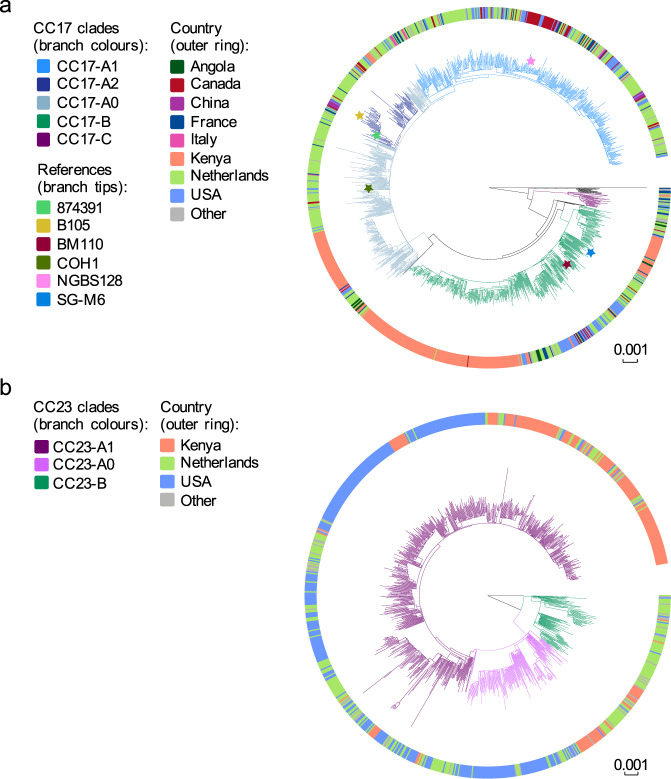
Global phylogeny of CC17 and CC23. Tree branches of each tree are coloured in accordance with CC clade assignment. Each tree is annotated with colour strips representing isolate’s country of origin (unique colour for countries with at least 10 isolates, ‘Other’: countries represented by less than 10 isolates). Scale bars represent the number of nucleotide substitutions per site. (**a**) a maximum likelihood phylogenetic tree of 1254 CC17 isolates from the Netherlands and other countries, including six reference genomes (reference branch tips marked with a star): NGBS128, COH1, B105, 874391, BM110 and SG-M6. (**b**) a maximum likelihood phylogenetic tree of 873 CC23 isolates from the Netherlands and other countries.

To determine if the CC17-A1 and CC23-A1 clones are specific to the Dutch GBS population or globally distributed, we combined our sequence data with the published genomes of CC17 (n = 722) and CC23 (n = 634) (Supplementary Data S2)^14,18,20–25^ and reconstructed a global phylogeny of each lineage (Fig. 5). For CC17, we also included six publicly available reference genomes.

The external dataset of CC17 contained isolates from 19 different countries although only seven countries were represented by more than 10 isolates (Supplementary Data S2). A total of 340 (47%) and 354 (49%) non-Dutch isolates represented clades CC17-A and CC17-B, respectively. More than half of global CC17-A isolates belonged to clone CC17-A1 (191/340; 56%), followed by CC17-A0 (116/340; 34%) and CC17-A2 (33/340; 10%). The CC17-A1 isolates were found in all external collections included in this analysis. The CC17-A1 clone was common among CC17 isolates from Canada (56/77, 73%), Italy (11/13, 85%), China (7/14, 50%), USA (70/159, 44%) and France (24/58, 41%). The reference genomes were distributed across the phylogeny, with the NGBS128 strain^20^ representing CC17-A1 and the B105 and 874391 strains representing CC17-A2 (Fig. 5).

The global dataset of CC23 genomes was represented largely by isolates from Kenya and the USA (Fig. 5). The non-Dutch samples were dominated by isolates from the CC23-A1 clade (480/636; 75%), which represented 75% of CC23 isolates from the USA (275/367) and 82% of CC23 isolates from Kenya (180/219).

### Expansion of the CC17-A1 clone correlates with acquisition of a novel phage

To investigate if expansion of the CC17-A1 clone can be attributed to presence of unique genetic markers, we investigated the core and accessory genome characteristics of the CC17-A1 cluster. Amongst core genome single nucleotide polymorphisms (SNPs) associated with the CC17-A1 clone (Supplementary Table S1), of particular interest is a double non-synonymous mutation in the *ciaH* gene (positions 954832 and 954833 in the *S. agalactiae* COH1 reference genome), which encodes the CiaH sensor histidine kinase. This mutation was found in all CC17-A1 isolates as well as 14 other CC17 isolates across the phylogeny (Supplementary Fig. S5), representing a homoplasic mutation that may indicate selective pressure acting on this locus.

**Figure 6:**
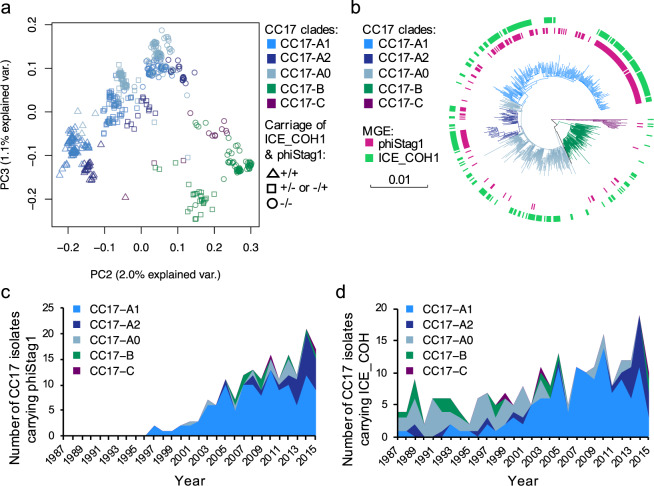
Distribution of phiStag1 and ICE_COH elements across CC17 population. (**a**) PCA of the accessory gene distribution in CC17. Each data point is colour-coded to show isolate clade assignment (CC17-A1, CC17-A2, CC17-A0, CC17-B or CC17-C). Data point shapes represent the pattern of ICE_COH1 and phiStag1 carriage to indicate if both MGEs are present (+/+), if only either one MGE is present (+/− or −/+), or if neither MGE is present (−/−). Isolates from CC17-A clade revealed all three patterns, while none of the CC17-B isolates carried both MGEs. (**b**) Distribution of ICE_COH1 and phiStag1 across the CC17 phylogeny. Tips of the phylogenetic tree are annotated with strips indicating presence of each MGE. Tree branches of are coloured in accordance with CC17 clade assignment. Scale bar represents the number of nucleotide substitutions per site. (**c**) Number of CC17 isolates carrying phiStag1 each year. (**d**) Number of CC17 isolates carrying ICE_COH each year.

To check if the CC17-A1 clone features a unique accessory genome we investigated the pan-genome of CC17 and clustered isolates based on gene presence/absence using principal component analysis (PCA). Most of the accessory gene variation within CC17 could be attributed to the difference between clades CC17-A and CC17-B (91% of variance explained by PC1). An additional subdivision was visualized by plotting PC2 against PC3, which revealed three clusters, each composed of isolates from the different CC17-A phylogenetic groups (Fig. 6). This subdivision was therefore not due to accessory genes unique to the CC17-A1 clone. Correlation analyses of the accessory gene presence/absence within these clusters showed that the clustering pattern was due to variable carriage of two sets of genes that were found to be associated with two different mobile genetic elements (MGEs). One was identified as an integrative conjugative element (ICE), described previously as ICE_COH1_tRNA^Lys^^26^ (hereafter referred to as ICE_COH1). This MGE encodes several virulence factors including antigen I/II^27^, CAMP factor II^28^, and manganese transporter MntH^29^. The second element was a phage, designated here as phiStag1 (also found in the NGBS128 reference genome, coordinates: 689741–748064). It was found to carry a gene (NGBS128 locus tag: AMM49_03725) encoding a putative cell-surface protein, containing an N-terminal YSIRK signal peptide, a C-terminal LPxTG cell-wall anchor motif and a L-type lectin domain with potential carbohydrate-binding properties.

Screening of all CC17 isolates showed that 45% (238/526) and 34% (179/526) carried ICE_COH1 and phiStag1 elements, respectively. Composition of PCA clusters reflected the distribution of these MGEs across the CC17 population, with isolates clustering based on carriage of both, either one or neither MGE (Fig. 6). Isolates carrying ICE_COH1 and phiStag1 were intermittently distributed across the CC17 phylogeny suggesting multiple horizontal acquisition events (Fig. 6). However, the prevalence of both MGEs was highest among CC17-A1 and CC17-A2 isolates. The phiStag1 was found in 60% (131/217) of CC17-A1 and 49% (28/57) of CC17-A2 isolates in contrast to just 7% (11/153) of CC17-A0 isolates. The CC17-A0 showed a higher prevalence of ICE_COH (56/153, 37%), although the element was still more common in CC17-A1 (130/217, 60%) and CC17-A2 (30/57, 53%) isolates. Analysis of temporal prevalence of the two elements among CC17 isolates showed that while ICE_COH1 occurred throughout the sampling period, isolates with phiStag1 were first detected in 1997 (Fig. 6), coinciding with the time of CC17-A1 clonal expansion. The number of CC17 phiStag1-positive isolates, predominantly from the CC17-A1 cluster, increased steadily from 2000.

Across the collection, phiStag1 occurred sporadically in isolates from other CCs, but no isolate carried phiStag1 before its first emergence within CC17 in 1997. The highest prevalence of phiStag1 outside of CC17 was among CC23 isolates (41/239, 17%), however, it was not overrepresented within the CC23-A1 cluster. As expansion of the CC23-A1 clone could not be attributed to carriage of phiStag1, a separate pan-genome analysis of the entire CC23 was conducted but this did not reveal any accessory genes or MGEs associated with the CC23-A1 clone.

## Discussion

Genomic analysis of a national GBS collection from cases of neonatal invasive disease in the Netherlands between 1987 and 2016 revealed expansion of historical clones from two major lineages: CC17 and CC23. A significant increase in isolates representing these clones, defined here as CC17-A1, CC17-A2 and CC23-A1, was sufficient to account for the overall increase in a number of GBS infection cases during this period. The largest single contributor to the increasing trend was the CC17-A1clone, and its clonal expansion was the key underlying mechanism for the previously reported rise in the number of CC17 isolates among cases of neonatal GBS infections in the Netherlands^11^. It also resulted in CC17 becoming the most prevalent GBS lineage each year from the mid-1990s up until the end of the study period. This further underscores the importance of CC17 as a dominant GBS lineage associated with neonatal disease and offers new insights into its population structure and changes over time. The expansion of CC23-A1 led to CC23 gradually becoming the second most common lineage, alongside CC19 isolates. The rise in the number of isolates representing the CC23-A1 clone was most significant among cases of EOD, which might be addressed by a better adherence to or a revision to IAP guidelines. In contrast, the CC17-A1 isolates increased significantly for both EOD and LOD cases, thus reviewing domestic IAP policy would only partly counteract the rising prevalence of CC17-A1-associated infections.

The driving forces behind the expansion of CC17-A1, CC17-A2 and CC23-A1 clones are unclear. A common driver of bacterial pathogen evolution is the antibiotic selective pressure which can to lead to clonal expansion of pre-existing bacterial genotypes that already carry resistance determinants^30^. Antimicrobial resistance may also play an important role in GBS epidemiology as expansion of a GBS clone showing macrolide resistance was recently reported in Portugal among isolates from invasive disease in non-pregnant adults^31^. There have also been reports of multidrug resistant clones of CC17 in Portugal and China that were found among isolates from neonatal disease^16,23^. Phenotypic antimicrobial susceptibility data was not available for our GBS collection and isolates were characterised by genotypic resistance typing only. Overall, we observed a low prevalence of macrolide resistance genes in this collection in comparison to macrolide resistance rates among GBS isolates from neonatal disease in other countries such as France and Portugal^3,31^. This might be due to a lower consumption of antibiotics in the Netherlands in comparison to other countries in Europe^32^. In the absence of phenotypic resistance data, genotypic resistance profiles might also underestimate the level of antimicrobial resistance in our GBS collection.

Genomic analysis did not reveal any association between the expansion of CC17 and CC23, and carriage of antimicrobial resistance determinants although the time of their clonal expansion coincided with the introduction of national GBS disease prevention guidelines in the Netherlands in 1999 that recommend antibiotic prophylaxis for women in labour with clinical risk factors for transmission of GBS disease. However, a link between IAP treatment and the rise in the frequency of isolates representing these GBS clones could not be established here as clinical data on whether mothers received the IAP was not available. It was therefore not possible to determine which disease cases were associated with a failed antimicrobial prophylaxis.

Expansion of pre-existing strains can be triggered by genetic changes within circulating clones such as acquisition of novel genes that alter bacterial virulence^33^. An increase in prevalence of a pre-existing GBS clone after it acquired new genetic determinants was recently reported for GBS ST-1 serotype V^34,35^. Our GBS collection showed an emergence of a novel phage phiStag1, which was first detected in a CC17-A1 isolate from 1997. This was followed by a year-on-year increase in a number of CC17 isolates carrying this phage, predominantly among the expanding CC17-A1 and CC17-A2 clones. The phiStag1 phage may have become more prevalent due to an association with a successful clone. Alternatively, the acquisition of phiStag1 directly contributed to the expansion of CC17-A1 and CC17-A2. Phages have been shown to play an important role in the evolution, emergence and persistence of virulent *S. pyogenes* lineages by facilitating acquisition of novel virulence traits^33,36^. In GBS, phage-associated genes represent around 10% of strain-specific genes and some might be involved in adaptation and virulence^37,38^. No resistance determinants were identified in the genome of phiStag1 but it was found to carry gene encoding a putative cell surface protein that could be involved in interaction with host factors. In parallel with our findings, acquisition of a gene encoding a novel cell-surface protein was linked to the expansion of the GBS ST-1 serotype V clone^35^, indicating that such genetic determinants may play an important role in the evolution of GBS virulence.

Analysis of genetic variants associated with the CC17-A1 clone also revealed a double non-synonymous mutation in the *ciaH* gene in all CC17-A1 isolates. Although this variant is not associated with a recent emergence as it occurred on a root of the CC17-A1 cluster, it represented a homoplasic mutation that was detected in other CC17 isolates and might therefore represent a pathoadaptive mutation. The *ciaH* gene encodes the CiaH sensor histidine kinase that belongs to the two-component regulatory system (TCRS) CiaRH. TCRSs have been shown to play an important role in GBS adaptation to changes in host environment, and can influence the fitness and pathogenesis of GBS^39–41^. Although it is currently unknown what environmental parameters are monitored by CiaH, the CiaR regulator has been associated with GBS intracellular survival and enhanced virulence in a mouse model of infection^42^.

Bayesian inference of phylogeny and past population changes showed that the sudden rise in the number of CC17-A1 isolates among disease cases correlated with a sharp increase in its relative genetic diversity and clonal expansion, which coincided with phiStag1 acquisition. This was not observed for CC23-A1, for which a clonal expansion event based on phylogenetic reconstruction occurred in the mid-1980s, a decade before it started to rise in frequency among GBS isolates from neonatal disease. Analysis of the CC23 pan-genome did not show association between CC23-A1 and any particular gene or a subset of genes. As such it is currently unclear which CC23-A1 genetic variants might have contributed to its recent rise in prevalence. It is possible that CC23-A1 represents a fitter and better adapted clone due to a particular composition of core genome SNPs.

We found that both CC17-A1 and CC23-A1 are globally distributed, however, a wider genomic surveillance of GBS is needed to determine their contribution to the burden of GBS invasive disease in neonates worldwide. As the scope of our work was limited to the analysis of isolates from disease it is unclear if the expanding CC17-A1 and CC23-A1 clones have also become more prevalent in carriage. Analysis of genomic diversity of GBS from maternal carriage is therefore required to better understand the association between the CC17-A1 and CC23-A1 clones and the invasive disease in neonates.

## Methods

### Bacterial isolates

Isolates were derived from a nationwide surveillance of bacterial meningitis and infant bacteraemia conducted by the Netherlands Reference Laboratory for Bacterial Meningitis (NRLMB). The NRLMB receives approximately 90% of cerebrospinal fluid (CSF) isolates of all patients with bacterial meningitis in the Netherlands^43,44^. In addition, they routinely receive blood isolates from infants with an invasive GBS infection from microbiology laboratories in the Netherlands^43^. We included isolates received between 01.1987 and 05.2016 that were collected from patients aged 0–89 days (n = 1345). Isolates were excluded if the date of birth or date of collection were not recorded, they were non-invasive, or if they were found to represent a second isolate from the second episode in a patient with more than one episode. Although the majority of isolates were derived from blood or cerebrospinal fluid, three samples were isolated from other body sites (Supplementary Data S1). Patient age was calculated as the number of days between the date of birth and the earliest known date of the illness; mostly the first date of culture. EOD was defined as invasive infection between 0 and 6 days after birth whereas LOD was defined as invasive infection between 7 and 89 days of life.

### Whole-genome sequencing, *de novo* genome assembly and annotation

Genomic DNA was extracted using Wizard® Genomic DNA Purification Kit from Promega. Tagged DNA libraries were created according to the Illumina protocol. Whole-genome sequencing was performed on the Illumina HiSeq. 2000 platform with 125 bp paired-end reads. Annotated assemblies were produced using a pipeline described previously^45^. For each sample, sequence reads were used to create multiple assemblies using VelvetOptimiser v2.2.5 (https://github.com/tseemann/VelvetOptimiser) and Velvet v1.2^46^. An assembly improvement step was applied to the assembly with the best N50 and contigs were scaffolded using SSPACE^47^, with sequence gaps filled using GapFiller^48^. Automated annotation was performed using PROKKA v1.11^49^ and a *Streptococcus*-specific database from RefSeq^50^.

### Molecular typing

Isolates were assigned to a CC group based on MLST^13^, hierBAPS clustering^51^ and core-genome phylogeny data. MLST was performed using SRST2^13,52^. For hierBAPS analysis a core genome SNP alignment was used that was generated using a reference-based approach. For this, sequence reads were mapped against the NGBS128 reference genome^20^ using SMALT v0.7.4 (https://www.sanger.ac.uk/science/tools/smalt-0) and SNPs were called using SAMtools v0.1.19^53^ and bcftools v0.1.19. Regions representing putative MGEs were masked using remove_blocks_from_aln (https://github.com/sanger-pathogens/remove_blocks_from_aln) and SNPs were identified with SNP-sites^54^. Alignment positions with >5% uncalled variants were filtered out. This core genome alignment was also used to reconstruct an approximately maximum-likelihood tree using FastTree^55^. Using MLST and hierBAPS clustering data isolates were assigned to a CC group if they represented the same hierBAPS cluster (except for the bin cluster) as the founder ST (confirmed with eBURSTv3^56^). The CC assignment was confirmed by plotting clusters on a phylogenetic tree and visually inspecting if isolates from the same CC belong to a monophyletic cluster. Isolates representing hierBAPS bin group, a polyphyletic cluster of low-frequency genotypes, that represented a single ST and were phylogenetically distant from other STs/CCs had a CC defined based on their ST.

CPS serotype was determined by *in silico* PCR using previously described primers for the detection of serotypes Ia, Ib and II-IX^57,58^. Carriage of antimicrobial resistance genes was checked with SRST2^52^ using the ARG-ANNOT database^59^, supplemented with sequences of GBS resistance determinants described by Metcalf *et al*.^18^.

### Phylogenetic reconstructions

To reconstruct the phylogeny of each major CC, a core genome SNP alignment was used that was generated using a reference-based method as described above. CC-specific *S. agalactiae* reference genomes were used for mapping: SS1 (CC1)^35^, Sag37 (CC10), NGBS128 (CC17)^20^, H002 (CC19)^60^, NEM316 (CC23)^61^. In addition to masking of regions representing putative MGEs, each CC was also screened for presence of variable sites associated with recombination using Gubbins^62^. The observed impact of recombination on genetic variation varied between CCs. Based on the recombination to mutation ratio (r/m) it was highest in CC10 (r/m = 6), followed by CC1 (r/m = 4.53), CC19 (r/m = 1.08), CC23 (r/m = 0.87) and was the lowest in CC17 (r/m = 0.24). For each CC, variable sites due to recombination were removed from the genome alignment. As a result, size of the core genome varied between the CCs and consisted of 2499 SNPs for CC1, 4572 for CC10, 18595 for CC17, 11979 for CC19 and 6739 for CC23. Maximum likelihood (ML) phylogenetic tree of each CC was generated with RAxML v8.2.8^63^ based on a generalised time reversible model with GAMMA method of correction for among site rate variation and 100 bootstrap replications.

The ML phylogenies were partitioned using fastbaps^64^. The CC17 and CC23 phylogenies were additionally partitioned using a method by Prosperi *et al*.^65^ based on pairwise SNP distances and node reliability of ≥90% (bootstrap support). For this, increasing pairwise SNP distance thresholds were tested, ranging from 150 to 300, with increments of 10. To identify expanding clusters, for each threshold that resulted in unique clustering pattern resulting clusters were checked for temporal trend by measuring the slope of a regression line based on the number of isolates from a cluster over time. Clusters with the highest positive slope value were selected.

To reconstruct time-calibrated phylogenies of CC17 and CC23, each sequence in the CC-specific core genome alignment was annotated with the date of isolation based on day, month, and year. A correlation between root-to-tip distance and the date of sampling was checked using TempEst^66^. The estimated correlation coefficient was R^2^ = 0.455 and R^2^ = 0.389 for CC17 and CC23, respectively. Bayesian inference of phylogeny and past population dynamics was performed with BEAST 2^67^ under a GTR + Γ nucleotide substitution model. Strict clock and relaxed lognormal clock models were compared, in combination with the constant, exponential and Bayesian skyline population models. Runs involving the relaxed lognormal clock model failed to converge. The strict molecular clock gave the best fit when used with the Bayesian skyline model. The MCMC chains were run for 50 million generations, sampling every 1000 states. Log files from six independent runs were combined after removal of burn-in (5% of samples) using LogCombiner and analysed with Tracer v1.5. The effective sample size (ESS) was 360 and 3251 for CC17 and CC23, respectively. Maximum clade credibility (MMC) tree was generated with TreeAnnotator. All phylogenetic trees were annotated using Evolview^68,69^.

### Core genome SNPs in CC17-A1

To analyse core genome SNPs in CC17 isolates, sequence reads were mapped using SMALT v0.7.4 (https://www.sanger.ac.uk/science/tools/smalt-0) to the COH1 reference genome^14^, and variants were called using SAMtools v0.1.19^53^ and bcftools v0.1.19. Variants were grouped by CC17 clusters. Variants located in regions representing putative MGEs were excluded from the analysis.

### Pan-genome of CC17

Pan-genome analysis of CC17 was performed with Roary^70^ using 95% identity threshold for blastp with splitting of clusters that contain paralogs. PCA of the accessory gene content was performed in R v3.3.3. Genes that were correlated (based on Spearman’s rank correlation coefficient) with selected PCA clusters were identified and analysed further. To determine their genomic context, genes were mapped back to whole genome assemblies using blastn pairwise sequence alignments^71^. Sequence similarity searches of genomic fragments found to contain the accessory genes of interest were performed using the NCBI BLAST and GenBank nucleotide sequence database^72^. MGE carriage was checked by short read mapping with SRST2^52^ using 90% sequence coverage cut-off.

## Supplementary information

Supplementary Information.

Supplementary Information2.

Supplementary Information3.

## Data Availability

Sequence reads were deposited in the European Nucleotide Archive under study accession PRJEB14124. Individual isolate accession numbers are provided in Supplementary Data S1. GenBank accession numbers of reference genomes used in this study are: SS1: CP010867, Sag37: CP019978, NGBS128: CP012480, H002: CP011329, NEM316: AL732656, COH1: HG939456, B105: CP021773, 87439: CP022537, BM110: LT714196 and SG-M6: CP021869). To reconstruct global phylogenies of CC17 and CC23, we included published genomes of 722 CC17 and 634 CC23 isolates from studies by Da Cunha *et al*.^14^, Rosini *et al*.^21^, Seale *et al*.^22^, Teatero *et al*.^20^, Campisi *et al*.^23,24^, Almeida *et al*.^25^ and Metcalf *et al*.^18^. Individual isolate accession numbers are provided in Supplementary Data S2.
