# Supplementary material for: Increasing incidence of group B streptococcus neonatal infections in the Netherlands is associated with clonal expansion of CC17 and CC23
Source: Sci Rep. 2020 Jun 12;10:9539. doi: 10.1038/s41598-020-66214-3 (PMC7293262; doi:10.1038/s41598-020-66214-3)
Supplement: Supplementary file 1 — Supplementary Information. [file 41598_2020_66214_MOESM1_ESM.pdf]

**Supplementary Information for**

# Increasing incidence of group B streptococcus neonatal infections in the Netherlands is associated with clonal expansion of CC17 and CC23

Dorota Jamrozy[a*], Merijn W Bijlsma[b], Marcus C de Goffau[a], Diederik van de Beek[b], Taco W. Kuijpers[c,d], Julian Parkhill[a], Arie van der Ende[e,f], Stephen D. Bentley[1]

[a] Wellcome Sanger Institute, Wellcome Genome Campus, Hinxton, UK
[b] Department of Neurology, Academic Medical Center, University of Amsterdam, Amsterdam Neuroscience, Amsterdam, Netherlands
[c] Department of Immunopathology, Sanquin Research and Landsteiner Laboratory of the Academic Medical Centre, University of Amsterdam, Amsterdam, Netherlands
[d] Department of Paediatric Haematology, Immunology and Infectious Diseases, Emma Children's Hospital, Academic Medical Centre, Amsterdam, Netherlands
[e] Department of Medical Microbiology, Academic Medical Center, University of Amsterdam, Amsterdam Infection & Immunity, Amsterdam, Netherlands
[f] Netherlands Reference Laboratory for Bacterial Meningitis, Amsterdam, Netherlands

* Address correspondence to Dorota Jamrozy, dorota.jamrozy@sanger.ac.uk

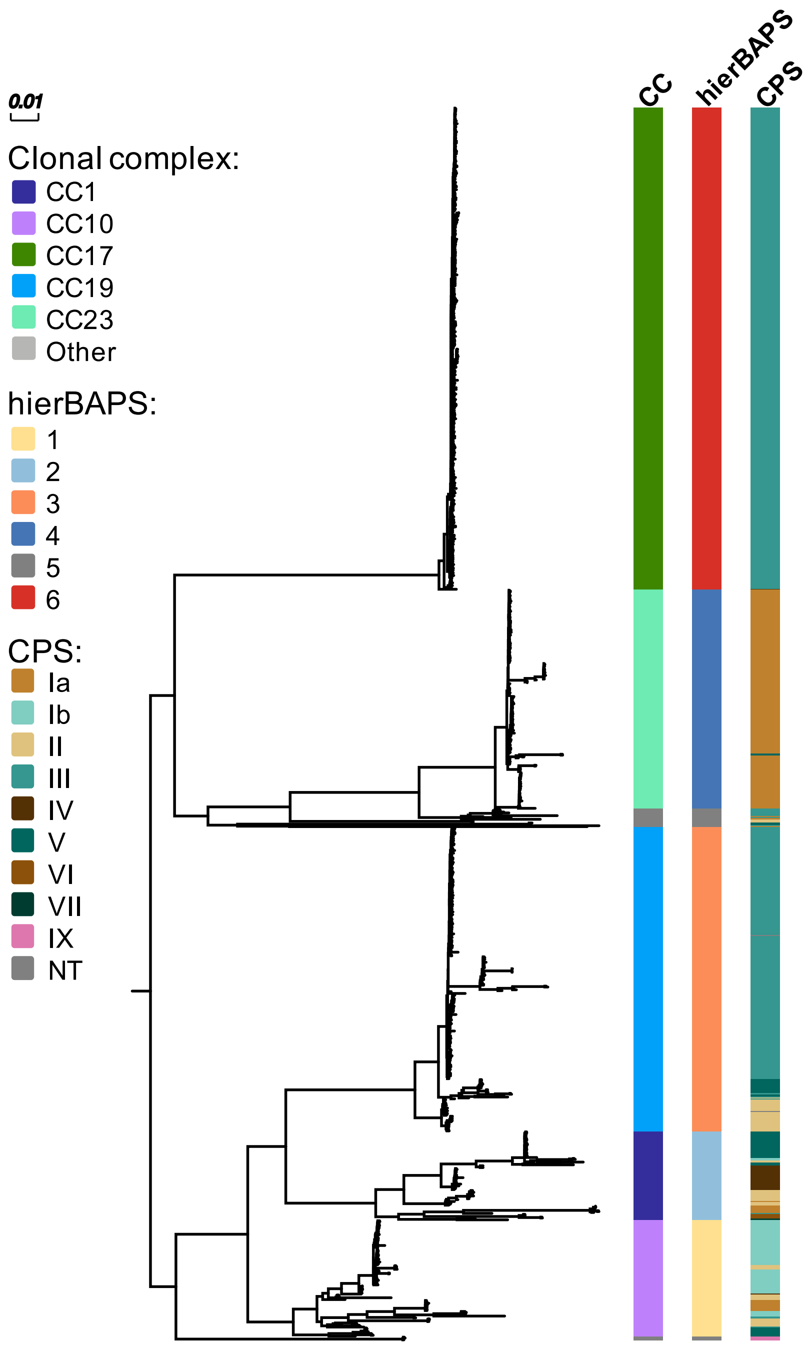


**Supplementary Figure S1**. An approximately maximum-likelihood phylogenetic tree of all GBS isolates. Tips of the tree are annotated with colour strips representing (from the left): clonal complex (CC), hierBAPS cluster ID and capsular polysaccharide (CPS) serotype. Scale bar the represents the number of nucleotide substitutions per site.

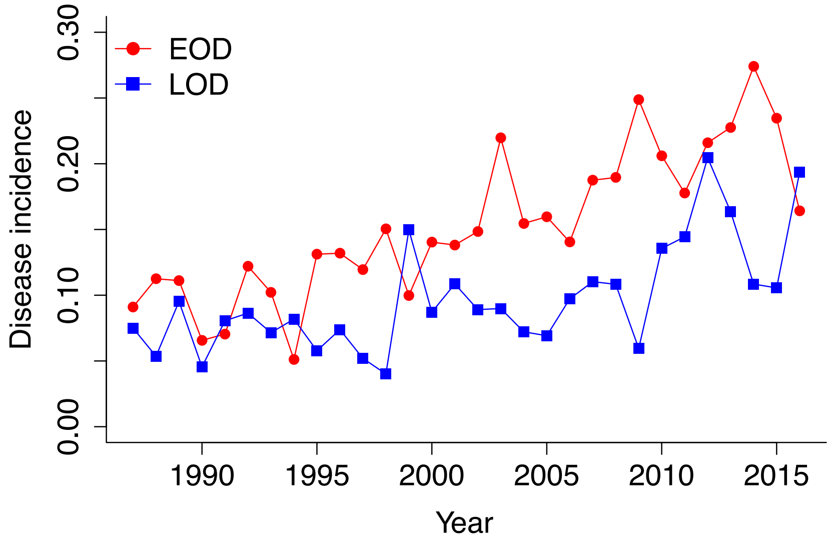


**Supplementary Figure S2.** Incidence of GBS invasive disease in infants aged 0 – 89 days in the Netherlands between 1987 and 2016. The incidence of early-onset disease (EOD) and late-onset disease (LOD) is shown as the number of cases per 1000 livebirths.

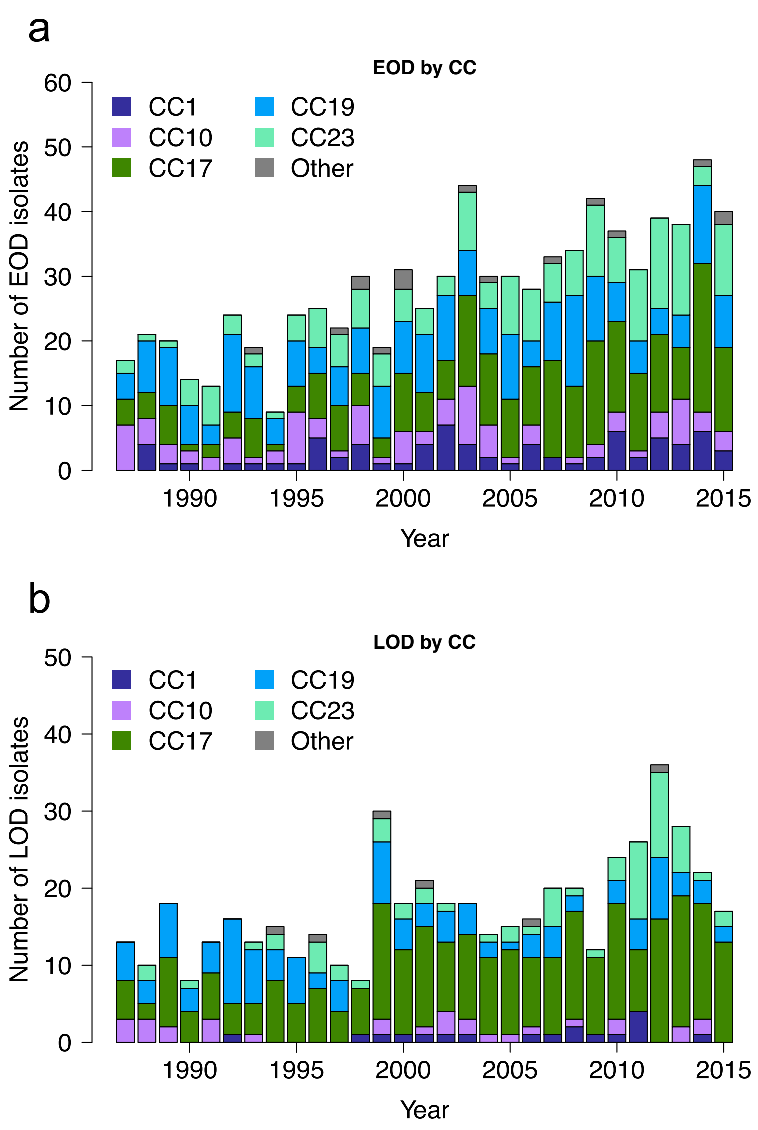


**Supplementary Figure S3.** Number of isolates from early-onset disease (EOD) and late-onset disease (LOD) each year stratified by clonal complex (CC). The exact number of isolates from each CC in each year is available in Supplementary Data 1. Number of isolates received in 2016 was excluded from the trend analysis as study sampling ended in May of that year. (a), EOD isolates. (b), LOD isolates.

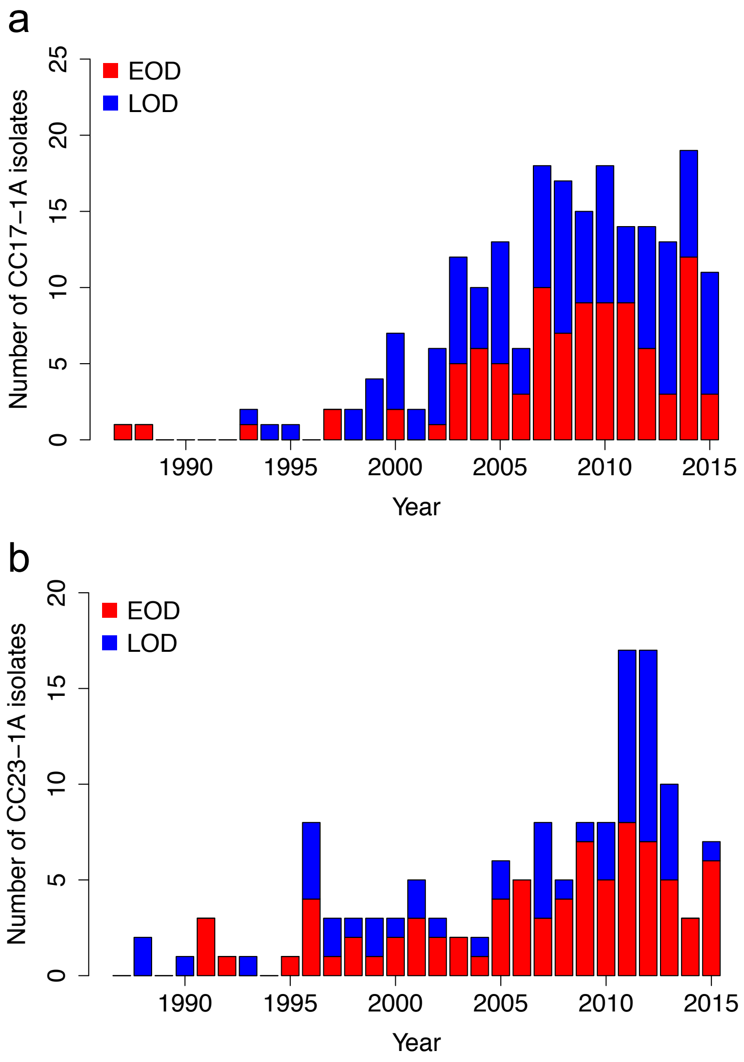


**Supplementary Figure S4**. Number of CC17-A1 and CC23-A1 isolates each year stratified by disease onset. The exact number of isolates from each clade in each year is available in Supplementary Data 1. Isolates from 2016 were excluded from the trend analysis as study sampling ended in May of that year. EOD: early-onset disease. LOD: late-onset disease. (a), CC17-A1 isolates (b), CC23-A1 isolates.

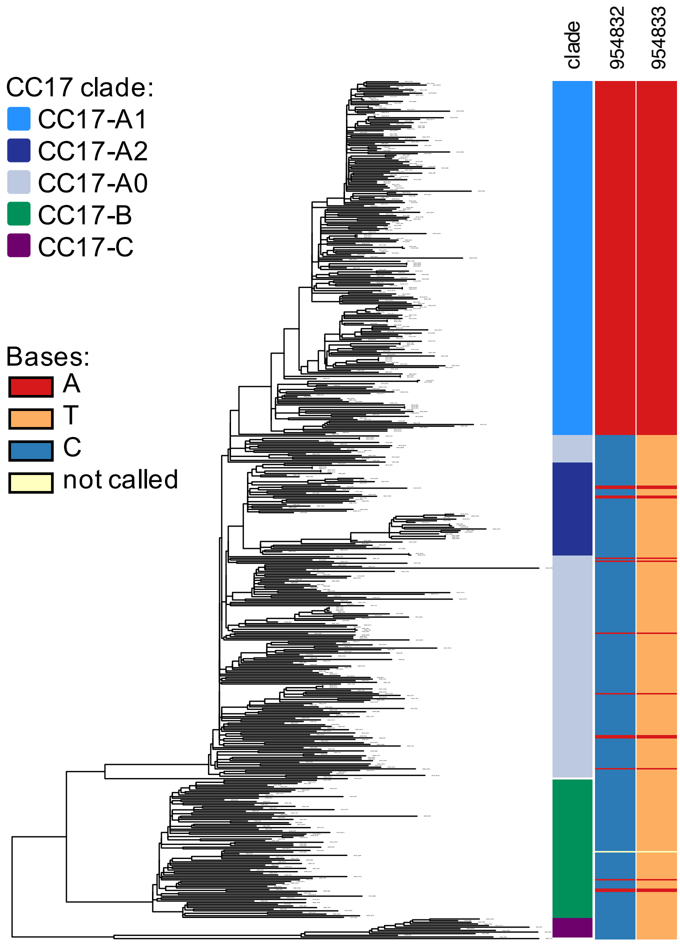


**Supplementary Figure S5**. Maximum-likelihood phylogenetic tree of CC17 and distribution of the *ciaH* gene SNPs. Tips of the tree are annotated with colour strips representing (from the left): CC17 clade, base variant at genomic positions 954832 and 954833 (within the COH1 reference genome).

**Supplementary Table S1.** List of SNPs associated with the CC17-A1 clade. Position: location in GBS COH1 reference genome (accession: HG939456). S/NS/I: synonymous/non-synonymous/intergenic. Ref: base at the locus in the reference genome. SNP: base at the locus in isolates with a SNP in comparison to the reference genome. Locus tag: name of the coding sequence in COH1 reference genome

| Position | S/NS/I | Ref | SNP | Locus tag | Product |
|---|---|---|---|---|---|
| 3585 | S | T | C | GBSCOH1_0003 | Diacylglycerol kinase catalytic domain protein, putative |
| 129999 | S | G | A | GBSCOH1_0118 | Ribose ABC transporter, ATP-binding protein |
| 226812 | S | G | A | GBSCOH1_0205 | Hypothetical protein |
| 384953 | S | A | G | GBSCOH1_0353 | ABC transporter, permease protein |
| 433411 | I | T | C | - | - |
| 457297 | N | G | A | GBSCOH1_0425 | Acetyltransferase, GNAT family |
| 602578 | N | C | A | GBSCOH1_0573 | Sortase family protein |
| 605291 | I | C | G | - | - |
| 954832 | N | C | A | GBSCOH1_0903 | Sensor histidine kinase CiaH |
| 954833 | N | T | A | GBSCOH1_0903 | Sensor histidine kinase CiaH |
| 959399 | S | G | T | GBSCOH1_0905 | Aminopeptidase N |
| 1258873 | N | C | T | GBSCOH1_1197 | ABC transporter, ATP-binding /permease protein |
| 1371010 | N | C | T | GBSCOH1_1301 | Large conductance mechanosensitive channel protein |
| 1394325 | N | C | A | GBSCOH1_1324 | Hypothetical protein |
| 1466238 | S | G | A | GBSCOH1_1392 | MTA /SAH nucleosidase |
| 1543780 | S | A | G | GBSCOH1_1476 | LemA protein |
| 1607198 | N | G | A | GBSCOH1_1541 | PTS system, IIABC components |
| 1654195 | N | C | T | GBSCOH1_1587 | ABC transporter, ATP-binding protein CydD |
| 1759676 | N | G | A | GBSCOH1_1690 | Sugar-binding transcriptional regulator RegR |
| 1799950 | N | G | A | GBSCOH1_1726 | Galactose-6-phosphate isomerase, LacA subunit |
